# Comparative and Transcriptome Analyses Uncover Key Aspects of Coding- and Long Noncoding RNAs in Flatworm Mitochondrial Genomes

**DOI:** 10.1534/g3.116.028175

**Published:** 2016-02-23

**Authors:** Eric Ross, David Blair, Carlos Guerrero-Hernández, Alejandro Sánchez Alvarado

**Affiliations:** * Howard Hughes Medical Institute; † Stowers Institute for Medical Research, Kansas City, Missouri 64110; ‡ College of Marine and Environmental Sciences, James Cook University, Townsville, Queensland, Australia

**Keywords:** Platyhelminthes, mitochondria, *Schmidtea mediterranea*, *atp8*, lncRNAs

## Abstract

Exploiting the conservation of various features of mitochondrial genomes has been instrumental in resolving phylogenetic relationships. Despite extensive sequence evidence, it has not previously been possible to conclusively resolve some key aspects of flatworm mitochondrial genomes, including generally conserved traits, such as start codons, noncoding regions, the full complement of tRNAs, and whether ATP8 is, or is not, encoded by this extranuclear genome. In an effort to address these difficulties, we sought to determine the mitochondrial transcriptomes and genomes of sexual and asexual taxa of freshwater triclads, a group previously poorly represented in flatworm mitogenomic studies. We have discovered evidence for an alternative start codon, an extended *cox1* gene, a previously undescribed conserved open reading frame, long noncoding RNAs, and a highly conserved gene order across the large evolutionary distances represented within the triclads. Our findings contribute to the expansion and refinement of mitogenomics to address evolutionary issues in this diverse group of animals.

The large phylum Platyhelminthes (flatworms) is an important member of the superphylum Lophotrochozoa (Simakov *et al.* 2013; Halanych *et al.* 1995). The Platyhelminthes is now regarded as consisting of a small group, the Catenulida, and its much larger sister, the Rhabditophora (Larsson and Jondelius 2008). Among the many major groups in the Rhabditophora is the Neodermata, containing the well-known parasitic groups, Monogenea, Trematoda, and Cestoda. Many complete mitochondrial (mt) sequences are available for neodermatans (Wey-Fabrizius *et al.* 2013) and, with the exception of some members of the genus *Schistosoma*, the mt gene order is generally conserved among these. Many studies on systematics, taxonomy, and biogeography of neodermatans have utilized data from mt genomes. Mitogenomic data for other taxa within the Rhabditophora are few, limiting options for study. Our interest is in the Tricladida, a major and widespread taxon with many representatives in marine, freshwater, and terrestrial habitats (Alvarez-Presas *et al.* 2008). To date, only two complete, and one near-complete, mt genome have been published for this taxon (Sakai and Sakaizumi 2012; (Solà *et al.* 2015). We present the complete mt sequences of the asexual (SmedAsxl) (Benazzi and Ballester 1970), and sexual (SmedSxl) (Benazzi *et al.* 1975) biotypes of the dugesiid triclad, *Schmidtea mediterranea*, as well as mt sequences from a *Girardia* species (Tricladida: Dugesiidae), and *Phagocata gracilis* (Tricladida: Planariidae)(Kenk *et al.* 1972). We use these and other available platyhelminth mt genomes, as well as transcriptional data, as guides for annotation. In addition, we identify a previously undescribed open reading frame (ORF), and uncover evidence for the transcription of long noncoding mt RNAs (lncmtRNA). Our analyses provide support for the existence of a TTG start codon, an extended *cox1* gene, and a broadly conserved mt gene order among the triclads.

## Materials and Methods

### Assembly of the mitochondrial genome sequence for a sexual biotype of S. mediterranea

**Figure 1:**
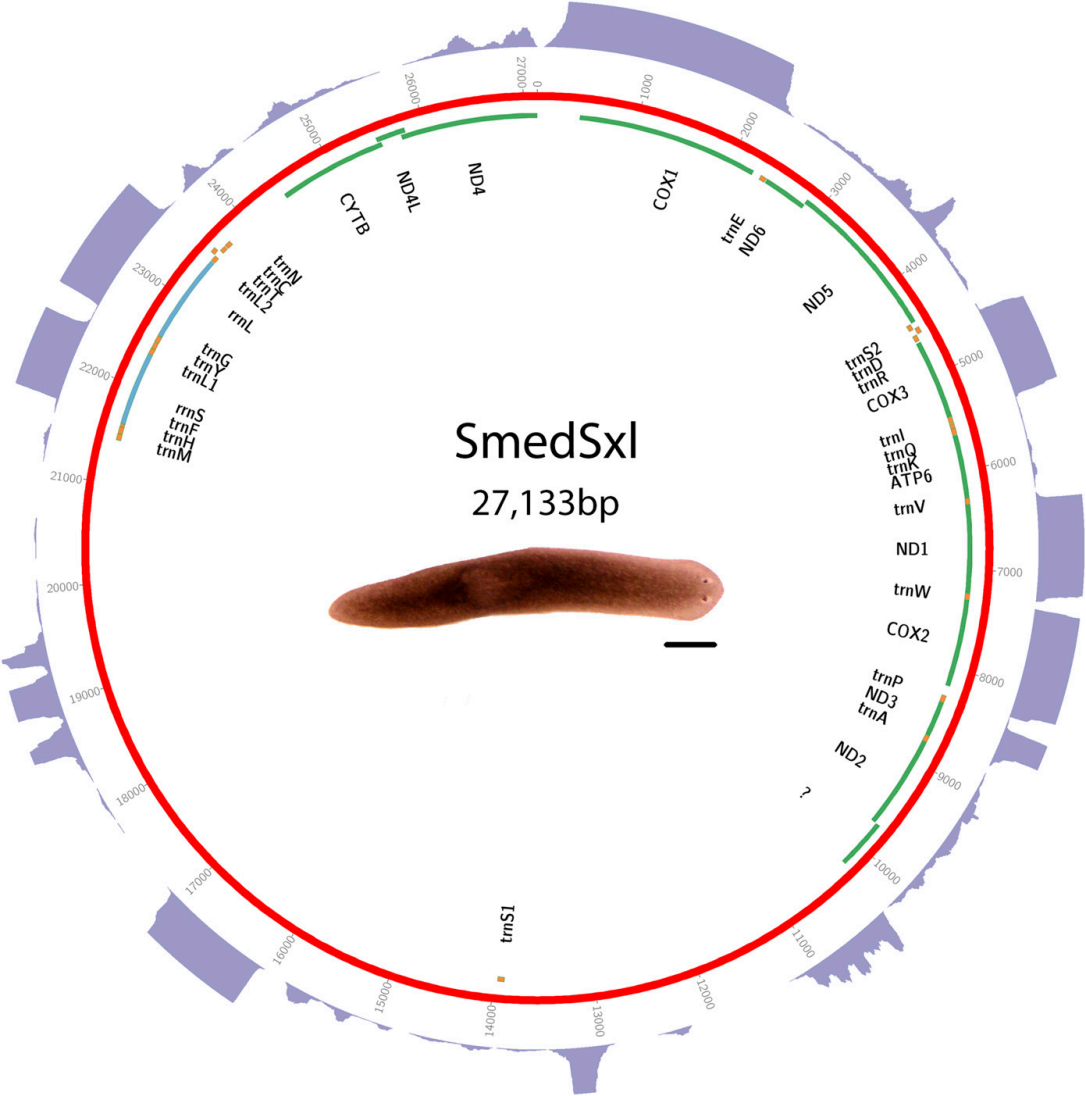
Schematic of *S. mediterranea* Sxl biotype mitochondrial genome. Putative protein-coding genes are in green. Putative ribosomal RNAs are in blue. tRNAs are in orange. The purple outer ring is a histogram representing depth of directional RNAseq aligned to mitochondrial genome. A maximum depth of 250 reads is displayed. Scale bar is 1 mm.

DNA was isolated from sexually mature and juvenile worms from a clonal hermaphroditic S2F2 strain of *S. mediterranea*. The sequencing was performed by the Genome Center at Washington University as part of the *S. mediterranea* genome project (Robb *et al.* 2008). Assembly was performed at the Joint Genome Institute. Sequence and annotations are available in GenBank as accession JX398125. See Figure 1 for a visual representation of the mt genome, and Supplemental Material, Table S1 for start and stop positions.

### Assembly of the mitochondrial genome sequence for an asexual biotype of S. mediterranea

**Figure 2:**
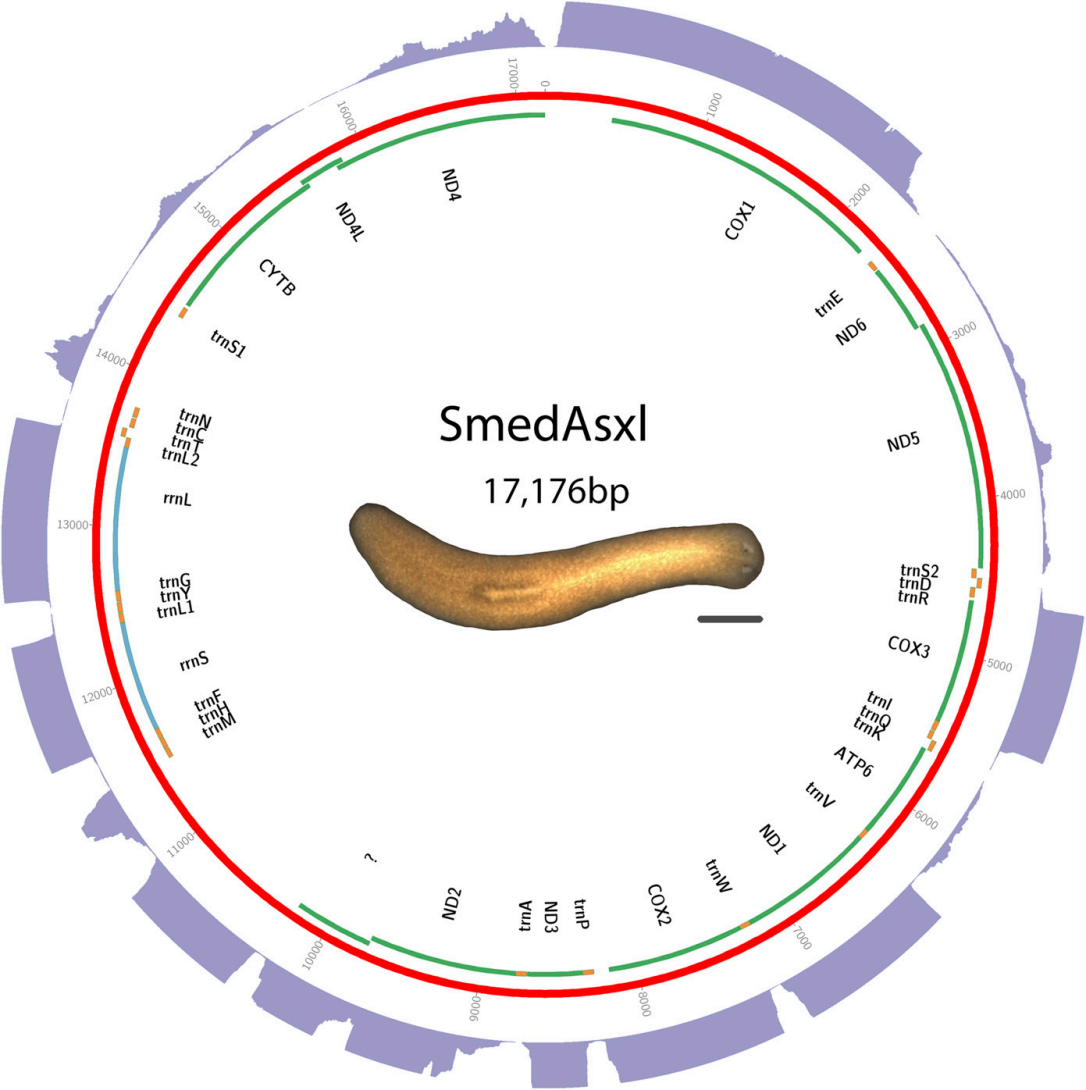
Schematic of *S. mediterranea* Asxl biotype mt genome. Putative protein-coding genes are in green. Putative ribosomal RNAs are in blue. tRNAs are in orange. The purple outer ring is a histogram representing depth of directional RNAseq aligned to mt genome. A maximum depth of 250 reads is displayed. Scale bar is 1 mm.

DNA was isolated from clonal worms of the asexual biotype of *S. mediterranea* (CIW4). Cofactor Genomics performed library construction and sequencing using the Illumina sequencing platform. Assembly was performed with SOAPdenovo2 (Luo *et al.* 2012) using a kmer size of 77. Other parameters were left at default. Sequence and annotations are available in GenBank as accession KM821047. See Figure 2 for a visual representation of the mt genome, and Table S2 for start and stop positions.

### PCR and sequencing of key regions of the S. mediterranea mitochondrial genomes

In order to validate the sizes of the assembled mitochondrial chromosomes of the sexual and asexual *S. mediterranea* strains, forward and reverse primers from regions predicted to be different were designed (Figure S5 and Table S5). Because of the repetitive nature of these sequences, and rich A/T content of these regions, annealing, extension, and amplification parameters were adjusted as follows. For sexual biotype, a melting temperature of 5° for 5 min followed by 35 rounds of amplification of 94° and 62° for 50 sec each, and 72° elongation for 5 min, was used. For the asexual biotype, all conditions remain the same except that only 20 rounds of amplification were required, and annealing and elongation temperatures were changed to 50° and 60°, respectively. The resulting products (Figure S6) were purified using QIAquick PCR purification (Qiagen) and bidirectionally sequenced, confirming the predicted sizes of the assembled *S. mediterranea* assembled genomes.

### Assembly of the mitochondrial genome sequence for an asexual biotype of Girardia sp.

**Figure 3:**
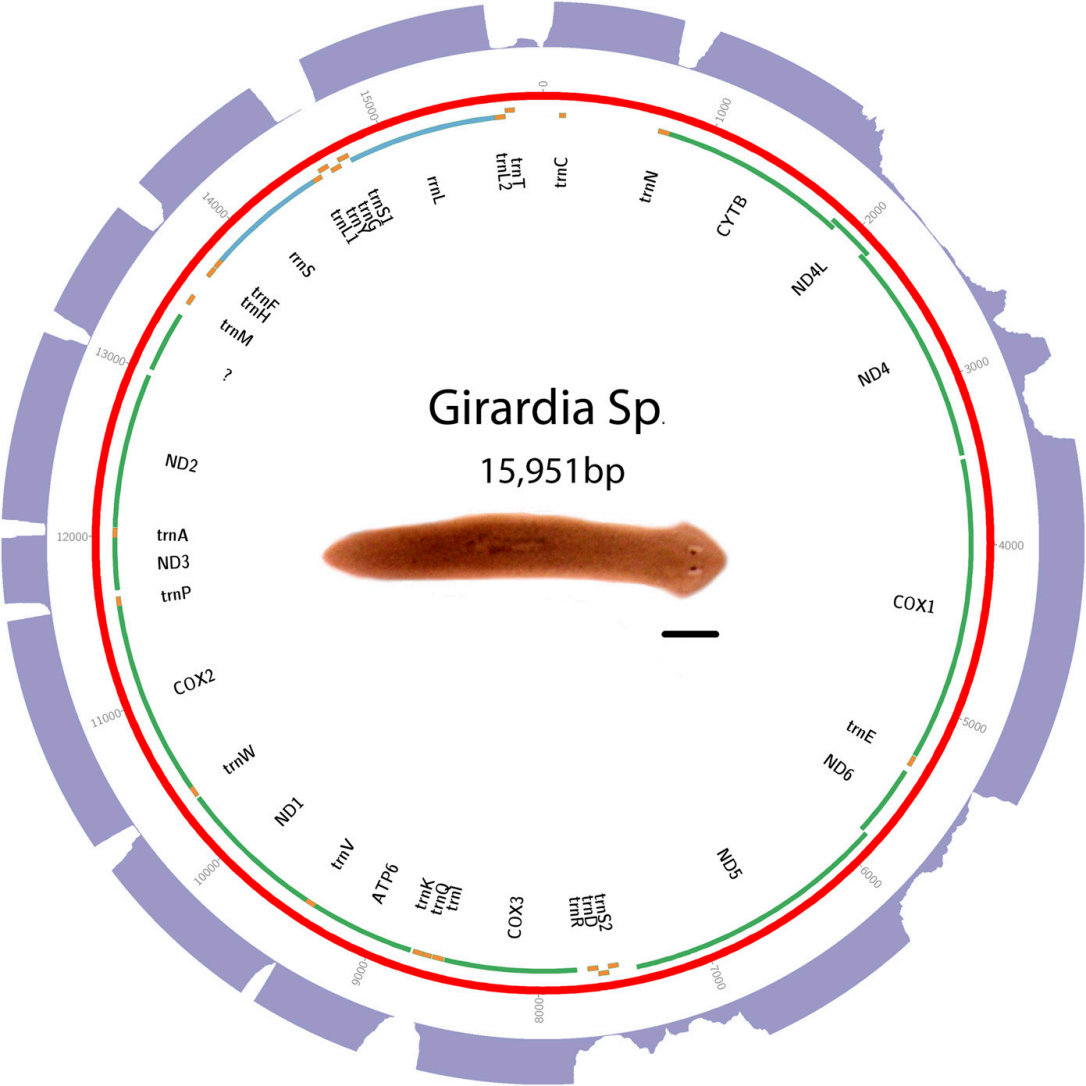
Schematic of *Girardia* sp. mt genome. Putative protein-coding genes are in green. Putative ribosomal RNAs are in blue. tRNAs are in orange. The purple outer ring is a histogram representing depth of RNAseq aligned to mitochondrial genome. A maximum depth of 250 reads is displayed. Scale bar is 1 mm.

DNA was isolated from clonal worms from an unknown *Girardia* species obtained from Ward’s Science as “brown planaria.” Library construction and sequencing was performed using the Illumina sequencing platform. Assembly was performed with SOAPdenovo2 (Luo *et al.* 2012) using a kmer size of 55. Other parameters were left at default. Sequence and annotations are available in GenBank as accession KP090061. See Figure 3 for a visual representation of the mt genome, and Table S3 for start and stop positions.

### Assembly of the mitochondrial genome sequence for an asexual biotype of P. gracilis

**Figure 4:**
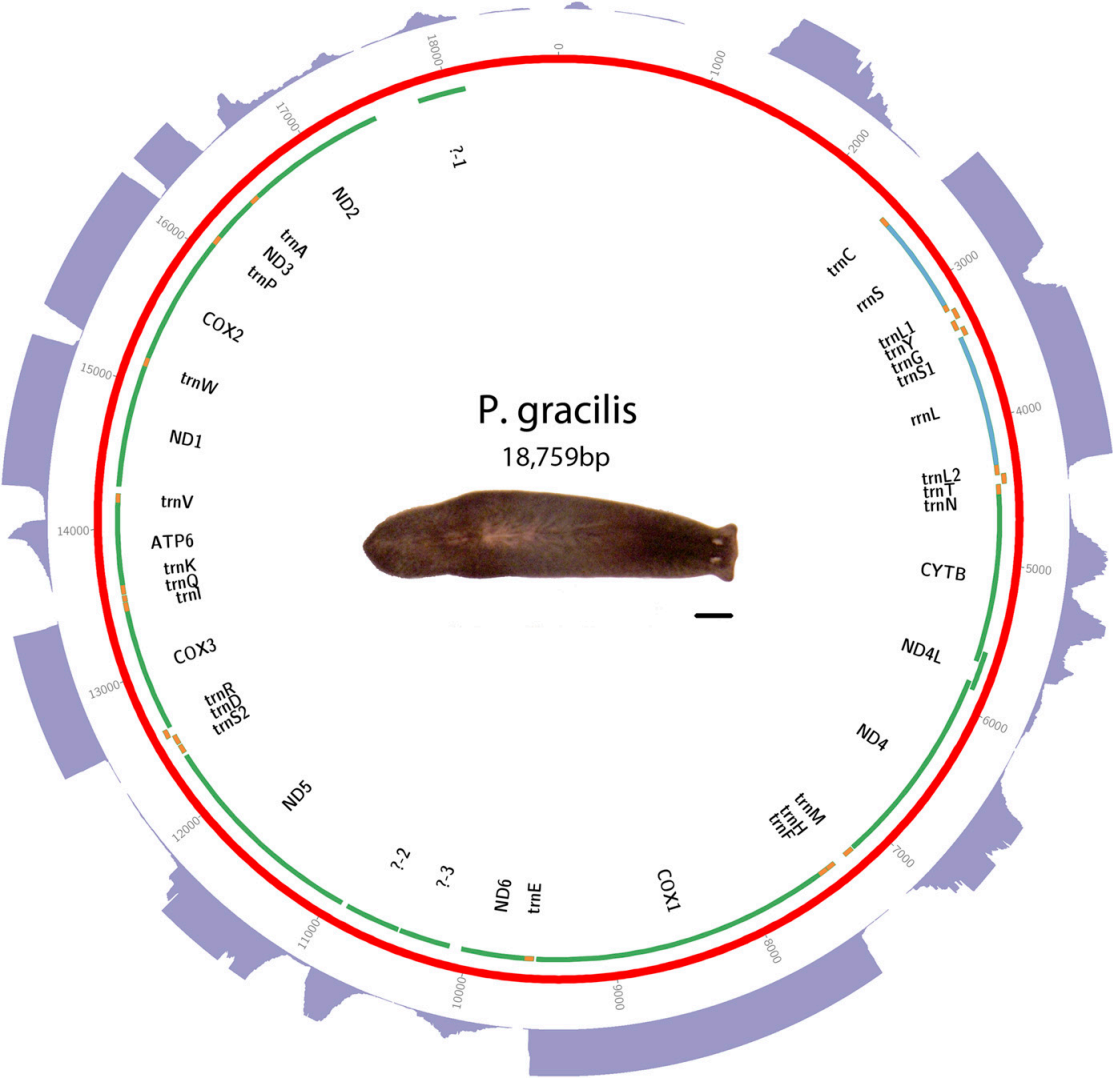
Schematic of *P. gracilis* mt genome. Putative protein-coding genes are in green. Putative ribosomal RNAs are in blue. tRNAs are in orange. The purple outer ring is a histogram representing depth of RNAseq aligned to mitochondrial genome. A maximum depth of 250 reads is displayed. Scale bar is 1 mm.

DNA was isolated from *P. gracilis* worms obtained commercially from Carolina Biological Supply as “black planaria”. Library construction and sequencing was performed using the Illumina sequencing platform. Assembly was performed with MITObim (Hahn *et al.* 2013). Sequence and annotations are available in GenBank as accession KP090060. See Figure 4 for a visual representation of the mt genome, and Table S4 for start and stop positions.

### RNA alignments to mitochondrial genomes

Uninjured adult planarians of the indicated species were pooled and homogenized in 600 μl of TRIzol Reagent (Ambion/Life Technologies). RNA isolation for each species was performed as indicated in the reagent manual. Briefly, phase separation using chloroform was performed, followed by RNA precipitation using isopropanol. The RNA pellets were washed with 75% ethanol and resuspended in 100 μl nuclease-free water to an approximate concentration of 550 ng/µl (*P. gracilis*), 491 ng/µl (*Girardia sp*.), 1566 ng/µl (*S. mediterranea*, asexual), and 764 ng/µl (*S. mediterranea*, sexual) . mRNAseq libraries were generated from 1 to 1.5 μg of high quality total RNA, as assessed using the Agilent 2100 Bioanalyzer. Libraries were made according to the manufacturer’s directions for the TruSeq RNA Sample Prep Kit v2, or TruSeq Stranded mRNA LT Kit (Illumina, RS-122-2101, and RS-122-2001). Resulting short fragment libraries were checked for quality and quantity using the Bioanalyzer. Equal molar libraries were pooled, requantified and sequenced as 100 bp paired reads on the Illumina HiSequation 2000 or 2500 instruments, using HiSeq Control Software 1.5.15 or 2.0.5, respectively. Following sequencing, Illumina Primary Analysis version RTA 1.13.48 or 1.17.20, and Secondary Analysis version CASAVA-1.8.2, were run to demultiplex reads for all libraries and generate FASTQ files. *S. mediterreanea* RNA was sequenced as directional paired-end reads. *Girardia* sp. and *P. gracilis* RNA was sequenced as nondirectional paired-end reads. Alignments were performed with bowtie2, using default parameters (Langmead and Salzberg 2012). Sequences are deposited with the NCBI (Accession Numbers:SmedSxl: SRR955511, SmedAsxl: SRR955099, *Girardia Sp*.: SRR319520, *P. gracilis*: SRR3194561).

### Transcript assembly and availability

The RNA sequencing described above was used to assemble transcripts for *Girardia* sp. and *P. gracilis*, using the Trinity transcriptome assembly tool with default parameters (Haas *et al.* 2013). Transcriptome sequences are already available for *S. mediterranea* (Robb *et al.* 2008).

### Mitochondrial gene annotations

The DOGMA (Wyman *et al.* 2004) and MITOS (Bernt *et al.* 2013) mt genome annotation tools were run on all genome assemblies to produce initial gene annotations. These annotations were additionally curated manually using homology and *de novo* transcriptome assemblies from each species to confirm start and stop positions. Circular diagrams were created with Circos (Krzywinski *et al.* 2009).

### Putative start codon position identification

In order to identify probable start codons for each of the 12 protein coding genes identified in *S. mediterranea*, we translated all flatworm mt genomes available in the NCBI, as well as our data for two biotypes for *S. mediterranea*, *P. gracilis*, and *Girardia* sp., into ORFs of at least 80 amino acids on the sense strand using EMBOSS (Rice *et al.* 2000). Translation Table 9 (European Nucleotide Archive, http://www.ebi.ac.uk/ena/browse/translation-tables), the echinoderm and flatworm mitochondrial translation table, was used. These ORFs were then aligned via BLASTP (Camacho *et al.* 2009) to our putative *S. mediterranea* gene annotations. The ORFs corresponding to each gene from each species were collected, and multi-aligned with MUSCLE (Edgar 2004).

### Data and reagent availability

All sequencing data are available in GenBank with accession numbers as listed in the respective sections of the *Materials and Methods*. Accessions for data deposited in public repositories are: GenBank JX398125, KM821047, KP090060, and KP090061.

## Results and Discussion

### Triclads contain the full complement of mitochondrial-encoded genes described in other Platyhelminthes

As previously reported for other species, mitochondrial sequences are well represented in extracts of genomic DNA. We took advantage of this to identify and assemble mt DNA and genomes of the SmedSxl and the SmedAsxl biotypes of *S. mediterranea*, *Girardia* sp., and *P. gracilis*. The relative abundances of mt sequences allowed us to generate assemblies of >6000×, >7000×, >2500×, >200× coverage for the SmedSxl, SmedAsxl, *Girardia* sp., and *P. gracilis* mt genomes, respectively (Figure 1, Figure 2, Figure 3, and Figure 4). The extensive coverage allowed us to annotate with confidence the genes encoded by these genomes including the tRNAs (Figure S1, Figure S2, Figure S3, and Figure S4). The genomes of *S. mediterranea*, *P. gracilis*, and *Girardia* sp., contain the full complement of mitochondrial genes reported for the phylum (Johnston 2006). Interestingly, we noticed a significant difference in the respective sizes of these mt genomes due to differing lengths of noncoding regions (Figure 1, Figure 2, Figure 3, and Figure 4). We conclude from these data that, even though the full complement of platyhelminth mt genes is found in the Planariidae, such similarity is not accompanied by comparable genome sizes.

### Directional RNA sequence supports that all mitochondrial transcription is from a single strand in triclads

We generated extensive directional transcriptional data for the *S*. *mediterranea* biotypes. Alignments of these to mt genomes were used to improve annotations. Because it has been previously reported that all mt genes of parasitic platyhelminths are transcribed from a single strand of DNA (Johnston 2006), we also sought to utilize our transcriptome data to test whether single-stranded transcription occurs in triclads. For both biotypes of *S*. *mediterranea*, except for a small number of sequences aligning antisense to the rRNA, all sequences aligning to the mitochondrial genomes aligned to the same DNA strand (Figure 1 and Figure 2). Given that the 16s RNA is a considerable contaminant in PolyA-selected mRNA, we suspect these rRNA alignments are not evidence of antisense transcription, but rather contamination of nonPolyA RNA (data not shown). We conclude from these data that transcription from a single strand of mitochondrial DNA is not an exclusive attribute of parasitic flatworms, but also shared by nonparasitic members of the phylum.

### Gene order is strikingly conserved in triclad mitochondrial genomes

**Figure 5:**
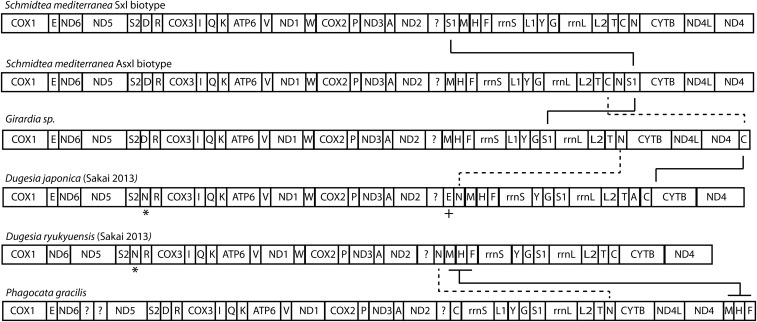
Schematic of gene order for six triclads, this includes five species and two biotypes of *S. mediterranea*. The tRNA marked “*” is a second Asparagine tRNA annotated in both *D. japonica* and *D. ryukyuensis*. It is unknown if this reflects a gene duplication, an annotation error, or some other change. The tRNA marked with a “+” is a second Glutamic acid tRNA annotated only in *D. japonica*.

In addition to the four genomes presented here, two other complete or near-complete triclad genomes have been published: *Dugesia ryukyuenis* and *Dugesia japonica* (Sakai and Sakaizumi 2012). These six genomes represent two superfamilies of triclads (Riutort *et al.* 2012) Despite the evolutionary distances between these species, very few mt gene rearrangements are apparent (Figure 5). For instance, there appears to be only a single tRNA difference between the *S. mediterranea* biotypes, two tRNA differences between the SmedSxl and *Girardia* sp., a single block of three tRNAs between *Girardia* sp. and *P. gracilis*, and two tRNAs rearranged between the Asxl *S. mediterranea* biotype and the two previously published *Dugesia* species (Figure 5). Moreover, some clusters of triclad mt genes are also seen in other Platyhelminthes. The gene cartridge composed of *cytb*, *nd4L*, and *nd4* is conserved in all studied parasitic platyhelminth mt genomes (Littlewood *et al.* 2006). Our data demonstrate the existence of this gene configuration in nonparasitic flatworms, and supports the hypothesis that such conservation of gene order may be a result of the extensive overlap of the genes composing this cartridge (Krakauer and Plotkin 2002).

### Noncoding regions in triclad mitochondrial genomes vary greatly in length

Even though the full complement of platyhelminth mt genes is present in triclads (Figure 1, Figure 2, Figure 3, and Figure 4), and the order of these genes is highly conserved (Figure 5), there is considerable variability in the length of the noncoding regions. At 27,133 bp in length, the mt genome of the SmedSxl biotype is exceeded in length by only 23 of the 4861 metazoan mt genomes deposited with NCBI (NCBI 2015), and it is by far the longest mt genome known from the Platyzoa (Wey-Fabrizius *et al.* 2013). Such extraordinary length is due to an extended long noncoding region (11,212 bp), interrupted only by a single putative *trnaS1* (Figure 1). In contrast, the *trnaS1* in the SmedAsxl biotype is found just before the *cytb* gene, and the long noncoding region is only 1249 bp. The unexpected size difference between the SmedSxl and SmedAsxl mt genomes and the physical location of *trnaS1* was confirmed via PCR (Figure S5, Figure S6, and Table S5). Amplification product sizes and sequences confirm the assembly. Interestingly, larger than expected products for SmedSxl lanes 5, 6, 7, and 8 in this AT rich, noncoding area of the genome, indicate that we may be underestimating the size of the SmedSxl biotype genome (Table S5). In *Girardia* sp., the long noncoding region is 861 bp in length, and is interrupted by a single tRNA (trnC), while the *P. gracilis* mt genome assembly displays a long noncoding region of 3726 bp.

Additionally, there are two putative and shorter noncoding regions in the *S. mediterranea* mt genomes ranging in size from 427 to 716 bp (Figure 1 and Figure 2). Between *nad4* and *cox1* there is a 427 bp noncoding region in both biotypes. This region does not appear to be conserved in *Girardia* sp., nor in the previously published triclad mitochondrial genomes (Sakai and Sakaizumi 2012). A second short putative noncoding region is found before the *cytb* gene. These regions are 716 bp and 639 bp in the SmedSxl and SmedAsxl biotypes, respectively. *P. gracilis* has a second short noncoding region of 321 bp immediately after *nad2*. We conclude that the diversity of lengths and positions of noncoding regions in triclad mt genomes may be under species- and/or strain-specific microevolutionary selective forces.

### New long noncoding RNAs may be transcribed from triclad mitochondrial genomes

Recently, long noncoding RNAs (lncRNAs) have been identified in what were previously thought to be noncoding regions in the human mt genome (Rackham *et al.* 2011). The depth of our RNAseq efforts allowed us to test whether lncRNAs may be transcribed from planarian mt genomes. Alignment of transcriptomes to their respective mt genomes identified several intergenic regions that appear to be transcriptionally active (Figure 1, Figure 2, Figure 3, and Figure 4). For instance, such regions were found in the SmedSxl mt genome at positions 16,100–17,099 bp (average depth of coverage ∼2000×), 23,740–24,449 bp (average depth ∼15×), and 18,500–19,599 bp (average depth ∼250×). Similarly, we also observed transcripts aligning to intergenic regions in *Girardia* sp. (positions 140–689; average depth ∼800×), and *P. gracilis* (positions 1269–2100; average depth ∼600×). The variability of depth coverage may reflect differences in RNA processivity, but this hypothesis needs to be tested experimentally. Nevertheless, because these transcripts are longer than 100 bp (Ørom *et al.* 2010), we conclude that mitochondrial long noncoding RNAs (mtlncRNAs) may be a characteristic of mt genomes across a broad phylogenetic distribution, and are deserving of further study.

### Noncanonical start codons are common in free-living flatworms

**Figure 6:**
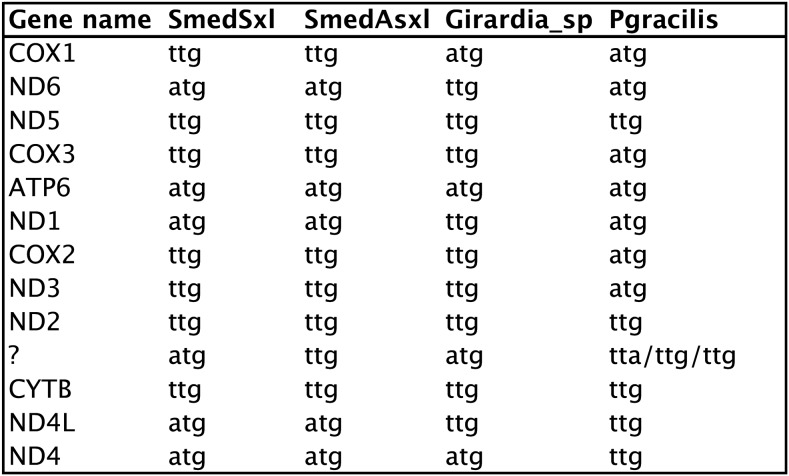
Predicted initiation codons for four triclad mitochondrial genomes. The three start codons for the *P. gracilis* are for each of three ORFs without homology.

Comparative alignments between ORFs of triclad species reveal TTG as a likely start codon for several genes (Figure 6). Manual examination of alignments identified highly conserved sequences extending beyond those predicted by the annotation engines. In many cases, similarity extended upstream beyond any possible ATG start, suggesting that an alternative initiation codon must be present. The most common codon that could resolve both homology, and minimize the distance between genes, was TTG (Figure 6, Table S1, Table S2, Table S3, and Table S4). Evidence for a TTG start codon has also been found in other triclads, including *D. japonica* (Solà *et al.* 2015). In addition to using homology to predict the most likely start codon positions, we examined RNAseq evidence. Figure 1, Figure 2, Figure 3, and Figure 4 show several positions with drops in the abundance of RNAseq, suggesting cleavage sites. These do not always correspond to annotated start codons, particularly in the case of *cox1*. In fact, in several cases these positions correspond with precision to TTG starts (*e.g.*, *cox2* and *nd3* in the SmedSxl mt genome; Figure 1 and Figure 6). The addition of a TTG to Translation Table 9 (http://www.ebi.ac.uk/ena/browse/translation-tables) should be considered for future mt genome annotations.

### Triclad mt genomes encode for an atypically large cox1 gene

**Figure 7:**
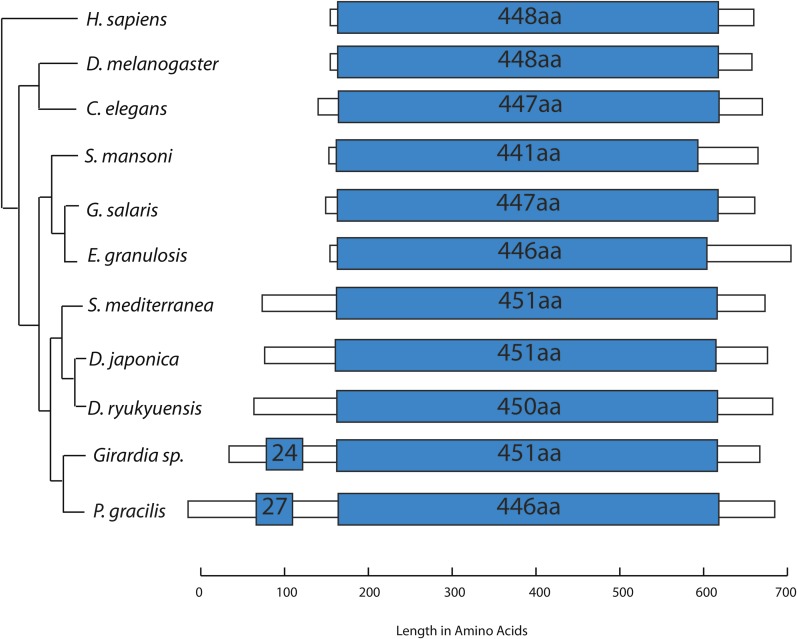
Cartoon of relative lengths of cytochrome *c* oxidase subunit 1 (COX1) mitochondrial-encoded genes from triclads, parasitic flatworms, human, and fly. Blue bars show the location and length of the conserved COX1 domain. *D. japonica*, and *D. ryukyuensis* COX1 genes are represented as the longest possible ORF rather than the existing annotation as the start position is unknown. The *cox1* gene labeled *S. mediterranea* represents both biotypes. Phylogenetic tree adapted from (Alvarez-Presas *et al.* 2008) and (Wägele and Bartholomaeus 2014).

Cytochrome *c* oxidase subunit 1 (COX1) is a highly conserved protein in metazoans, both in amino acid sequence and in size (Folmer *et al.* 1994). The C-terminus of the triclad COX1 proteins contains the highly conserved, canonical cytochrome c/quinol oxidase polypeptide I domain (PFAM 00115, e-value < 1e–100) (Figure 7); however, its precise identification in the triclad mt genomes was challenging even after considering TTG start codons. The ORFs coding for COX1 were longer than expected in each of the analyzed triclad mt genomes due to the presence of an extended 5′ end (N-terminus). The average length of the triclad *cox1* ORFs is 630 aa. The shortest is *D. ryukyuensis* at 621 aa, and the longest is *P. gracilis* at 700 aa (Figure 7). This is in contrast to the conserved size of *cox1*, not only in sister metazoan groups, such as the deuterostomes (*Homo sapiens*, 513 aa) and the ecdysozoans (*Drosophila melanogaster*, 483 aa; *Caenorhabditis elegans*, 525 aa), but also in evolutionarily distant organisms such as plants (*Arabidopsis thaliana*, 527 aa). The average length of the *cox1* genes currently deposited in REFSEQ for the Metazoa is 516 aa. In addition to those species reported in this paper, only two species have NCBI-deposited *cox1* genes longer than 600 aa (the coral *Euphyllia ancora* at 694 aa, and the sponge *Plakena trilopha* at 766 aa) (NCBI 2015). We conclude that an unusually long *cox1* gene is characteristic of triclads.

### Transcriptomic analyses revealed a transcribed ORF that may represent a noncanonical atp8

**Figure 8:**
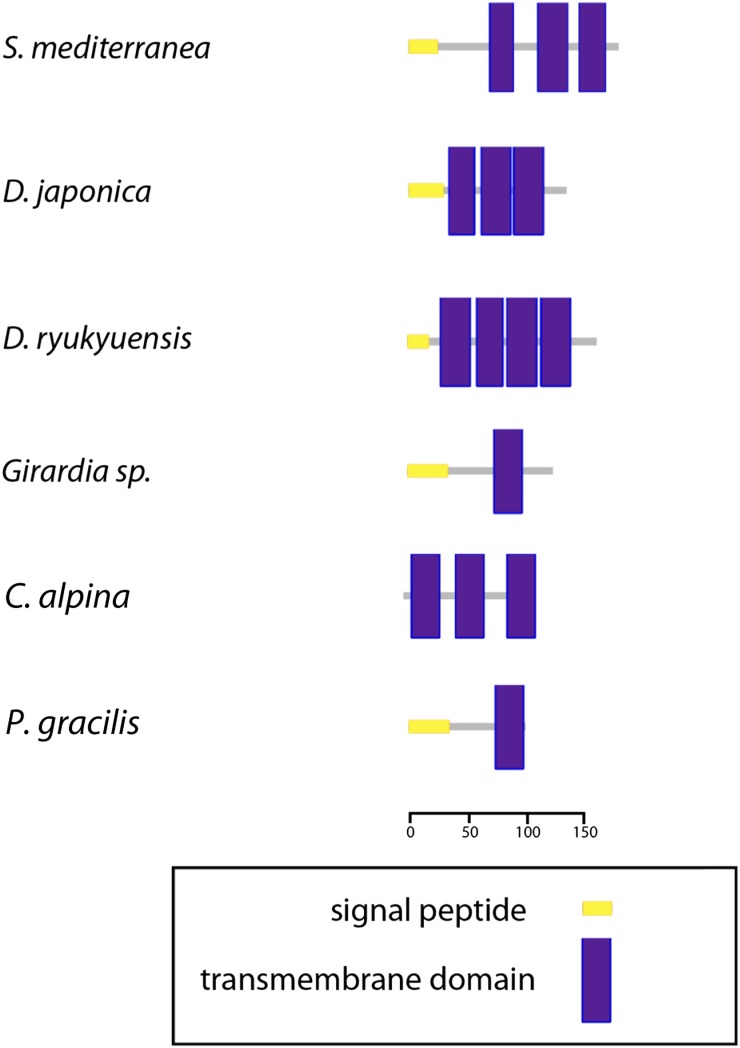
Cartoon of transmembrane domains and signal peptides as predicted by SMART for the ORFs immediately following ND2 in triclad mitochondrial genomes.

After aligning the transcriptome to the mt genomes of *Girardia* sp., *P. gracilis*, and *S. mediterranea*, we noticed a previously undescribed transcript mapping directly after *nad2* in both species (Figure 1, Figure 2, Figure 3, and Figure 4). These novel transcripts contained a *bona fide* ORF in all three species. *P. gracilis* contains two other ORFs between nd5 and nd6 as well; these are not conserved in position. Remarkably, inspection of three other published flatworm mitochondrial genomes, *i.e.*, *D. ryukyuensis*, *D. japonica* (Sakai and Sakaizumi 2012), and *Crenobia alpina* (Solà *et al.* 2015) uncovered sequences of similar lengths with predicted ORFs immediately following *nad2*. The canonical *ATP8* in Metazoa contains both a signal peptide and transmembrane domain, but the sequence is poorly conserved beyond the first four amino acids, MPQL (Hoffmann *et al.* 1992). Interestingly, like the canonical ATP8, each ORF is predicted by SMART (Letunic *et al.* 2014) to contain at least one transmembrane domain and, with the exception of *C. alpina*, signal peptides (Figure 8). Because of its conserved location and predicted motif architecture, we postulate that this transcriptionally identified ORF may code for a highly divergent triclad ATP8.

## Discussion

Traditionally, the annotation of mt genomes involves gene prediction tools and is based upon homology to other species. Although this approach has been very successful in bringing order to the wealth of mt genome sequences available (NCBI 2015), this strategy has some limitations. For instance, poorly conserved genes may be undetected, and, in the absence of an experimentally established translation table for the organisms under study, gene prediction efforts may result in either truncated or missing gene annotations. We sought to overcome these difficulties by taking advantage of the depth of data that is routinely acquired during RNA sequencing experiments. Such data sets normally encompass mitochondrial transcripts that should, in principle, complement traditional mt genome annotation efforts.

The abundance and quality of RNAseq reads that mapped to mt genomes was remarkable (Figure 1, Figure 2, Figure 3, and Figure 4). The depth of the sequence was more than sufficient to map to the full complement of predicted mt genes described for triclads, providing, for the first time for any taxon, systematic transcriptional evidence to support the annotation of newly assembled mt genomes. Remarkably, this method identified novel transcripts that mapped to mt genomic regions not previously known to be transcriptionally active. For instance, we discovered a putative mtlncRNA in *S. mediterranea*. In vertebrates, mtlncRNAs have been described both antisense to genes as well as in noncoding regions (Rackham *et al.* 2011). This novel class of mt transcripts, therefore, occurs in taxa other than mammals, and merits further investigation. Although it is unclear what the biological importance of these may be, recent evidence from mammals indicates that mtlncRNAs may be associated with aging endothelial cells (Bianchessi *et al.* 2015).

In addition to the identification of a mtlncRNA, we identified an ORF that may be a triclad ATP8. This has been identified as a vital component in mitochondrial ATP synthase in yeast and is presumed to operate similarly in Metazoa (Stephens *et al.* 2003). The absence of ATP8 in the mt genomes of several taxa, including Platyhelminthes, and other platyzoans (Wey-Fabrizius *et al.* 2013), has long been unexplained (Gissi *et al.* 2008). Without functional experiments, it is impossible to definitively label this transcribed ORF as *atp8*, but we believe that it is more parsimonious that a conserved ORF be a gene known to be present in other mitochondrial genomes, rather than a member of an entirely new class of genes. Future biochemical studies in heterologous systems should be able to address this possibility.

The ability to integrate RNAseq as part of mt genome assembly pipelines not only allows for improved gene annotation, but also creates novel opportunities for gene discovery. Our findings that many flatworm mt genomes transcribe both lncRNAs, and a positionally conserved ORF, demonstrate the value of this approach. These novel data not only further progress toward resolving long-standing issues with flatworm mt genomes, but also indicate that this approach can be used to shed light on previously unknown transcriptional activities of extranuclear DNA. The results reported here emphasize that the biological functions of mt genomes are understudied, and that the addition of RNAseq to the mt genome analysis toolkit is a step forward toward resolving this deficiency.

## Supplementary Material

Supplemental Material
