# Supplementary material for: Comparative and Transcriptome Analyses Uncover Key Aspects of Coding- and Long Noncoding RNAs in Flatworm Mitochondrial Genomes
Source: G3 (Bethesda). 2016 Feb 23;6(5):1191–200. doi: 10.1534/g3.116.028175 (PMC4856072; doi:10.1534/g3.116.028175)
Supplement: Supplemental Material [file supp_6_5_1191__index.html]

Comparative and Transcriptome Analyses Uncover Key Aspects of Coding- and Long Noncoding RNAs in Flatworm Mitochondrial Genomes — Supplemental Material 

# Comparative and Transcriptome Analyses Uncover Key Aspects of Coding- and Long Noncoding RNAs in Flatworm Mitochondrial Genomes

## Supplemental Material for Ross *et al.*, 2016

**Files in this Data Supplement:**

- Figure S1 - Trna figures S. mediterranea Sxl. (.pdf, 521 KB)
- Table S4 - *P. gracilis* Feature Table. (.pdf, 78 KB)
- Table S5 - Primers for PCR and sequencing of key regions of the *S. mediterranea* mitochondrial genome. (.pdf, 311 KB)
- Figure S2 - Trna figures S. mediterranea Asxl. (.pdf, 490 KB)
- Figure S3 - Trna figures Girardia sp. (.pdf, 332 KB)
- Figure S4 - Trna figures P. gracilis. (.pdf, 302 KB)
- Figure S5 - Schematic of PCR and sequencing of key regions of the *S. mediterranea* mitochondrial genome. (.pdf, 369 KB)
- Figure S6 - Gel of PCR of key regions of the *S. mediterranea* mitochondrial genome. (.pdf, 311 KB)
- Table S1 - SmedSxl Feature Table. (.pdf, 202 KB)
- Table S2 - SmedAsxl Feature Table. (.pdf, 202 KB)
- Table S3 - *Girardia sp.* Feature Table. (.pdf, 281 KB)
