# Supplementary material for: Comparative and Transcriptome Analyses Uncover Key Aspects of Coding- and Long Noncoding RNAs in Flatworm Mitochondrial Genomes
Source: G3 (Bethesda). 2016 Feb 23;6(5):1191–200. doi: 10.1534/g3.116.028175 (PMC4856072; doi:10.1534/g3.116.028175)
Supplement: Supplemental Material [file supp_g3.116.028175_TableS4.pdf]

Table S4 – P. gracilis Feature Table

Name	Start	Stop	Length	Distance	Putative Start Codon
trnC(gca)	2449	2516	68	3726	
rrnS	2517	3178	662	1	
trnL1(tag)	3172	3225	54	-6	
trnY(gta)	3226	3298	73	1	
trnG(tcc)	3299	3368	70	1	
trnS1(tct)	3362	3421	60	-6	
rrnL	3422	4320	899	1	
trnL2(taa)	4321	4391	71	1	
trnT(tgt)	4383	4451	69	-8	
trnN(gtt)	4453	4522	70	2	
CYTB	4514	5668	1155	-8	ttg
ND4L	5622	5870	249	-46	atg
ND4	5875	7200	1326	5	ttg
trnM(cat)	7208	7276	69	8	
trnH(gtg)	7364	7425	62	88	
trnF(gaa)	7429	7491	63	4	
COX1	7493	9595	2103	2	atg
trnE(ttc)	9547	9610	64	-48	
ND6	9611	10060	450	1	atg
unknown-3	10126	10476	351	66	ttg
unknown-2	10491	10871	381	15	ttg
ND5	11120	12379	1260	249	atg
trnS2(tga)	12403	12462	60	24	
trnD(gtc)	12472	12538	67	10	
trnR(tcg)	12539	12599	61	1	
COX3	12600	13437	838	1	atg
trnI(gat)	13438	13501	64	1	
trnQ(ttg)	13502	13555	54	1	
trnK(ctt)	13556	13620	65	1	
ATP6	13621	14193	573	1	
trnV(tac)	14179	14242	64	-14	
ND1	14291	15127	837	49	atg
trnW(tca)	15126	15187	62	-1	
COX2	15188	16081	894	1	atg
trnP(tgg)	16083	16145	63	2	
ND3	16146	16457	312	1	atg
trnA(tgc)	16458	16520	63	1	
ND2	16523	17482	960	3	ttg
unknown-1	17794	18122	329	312	tta
