# Supplementary material for: Comparative and Transcriptome Analyses Uncover Key Aspects of Coding- and Long Noncoding RNAs in Flatworm Mitochondrial Genomes
Source: G3 (Bethesda). 2016 Feb 23;6(5):1191–200. doi: 10.1534/g3.116.028175 (PMC4856072; doi:10.1534/g3.116.028175)
Supplement: Supplemental Material [file supp_g3.116.028175_TableS5.pdf]

Table S5 - Primers for PCR and sequencing of key regions of the *S. mediterranea* mitochondrial genome.

Gel	Lane	Fwd Primer	Rev Primer	Start	Stop	predicted insert	size from gel
SmedSxl	1	cgtgagtctcagtctgtgtatgagtgtggg	acaatccacaaacagactgaagaaaaaacc	8456	10172	1716	~1.7kb
SmedSxl	2	cgatttaaaatacgaactttggaggtttgag	ggttgtttatatgacgataataaactcgacc	8299	9989	1690	~1.6kb
SmedSxl	3	ggatgttgagttgtgttttgtattacagatgcc	ctttaacaatcaagaaaccaaaccaaaagacc	8560	9865	1305	~1.3kb
SmedSxl	4	gtgtttggtgtttgattagagtattttaaagg	ccaaaaagaattagaagtatgaaccattaagc	8657	9754	1097	~1.1kb
SmedSxl	5	ggttttttcttcagtctgtttgtggattg	caacaacatcaggttagagaccac	10143	13941	3798	~5kb
SmedSxl	6	ggtcttttggtttggtttcttgattgttaaag	caacaacatcaggttagagaccac	9834	13941	4107	~6kb
SmedSxl	7	atgttttccacacttctccctgtctc	catagcaatacaaagtgtaaccgcagaagctggc	13132	21843	8711	>12kb
SmedSxl	8	atgttttccacacttctccctgtctc	ctcaaaagaagcgccgaaacgaaagcggc	13132	21348	8216	>12kb
SmedAsxl	1	gtctgtttatgagtgtgggtttgattctaagg	ggaaaacctgtgagagtcacatacaatacc	8461	9484	1023	~1kb
SmedAsxl	2	ggttggatttgttgggttacaataggtttcg	cagcactaaagtcaaaaccaagtacataaccc	8421	9371	950	~1kb
SmedAsxl	3	ggatgttgagttgtgttttgtattacagatgcc	ggaaaacctgtgagagtcacatacaatacc	8554	9484	930	~1kb
SmedAsxl	4	gcaactttacattgtgcttgttattaggctcc	cagcactaaagtcaaaaccaagtacataaccc	8736	9340	604	~.6kb
SmedAsxl	5	gtctgtttatgagtgtgggtttgattctaagg	gctaccttcgtacagtcaaagtactgcagc	8461	13958	5497	~5.5kb
SmedSxl specific	Sxl	atgttttccacacttctccctgtctc	caacaacatcaggttagagaccac	13132	13941	809	~.8kb
SmedSxl specific	Asxl	atgttttccacacttctccctgtctc	caacaacatcaggttagagaccac	na	na	0	0
