# Supplementary material for: Comparative and Transcriptome Analyses Uncover Key Aspects of Coding- and Long Noncoding RNAs in Flatworm Mitochondrial Genomes
Source: G3 (Bethesda). 2016 Feb 23;6(5):1191–200. doi: 10.1534/g3.116.028175 (PMC4856072; doi:10.1534/g3.116.028175)
Supplement: Supplemental Material [file supp_g3.116.028175_FigureS2.pdf]

Figure S2 - Trna figures S. mediterranea Asxl

tRNA secondary structures as predicted by MITOS.

Schmidtea mediterranea - Asxl biotype

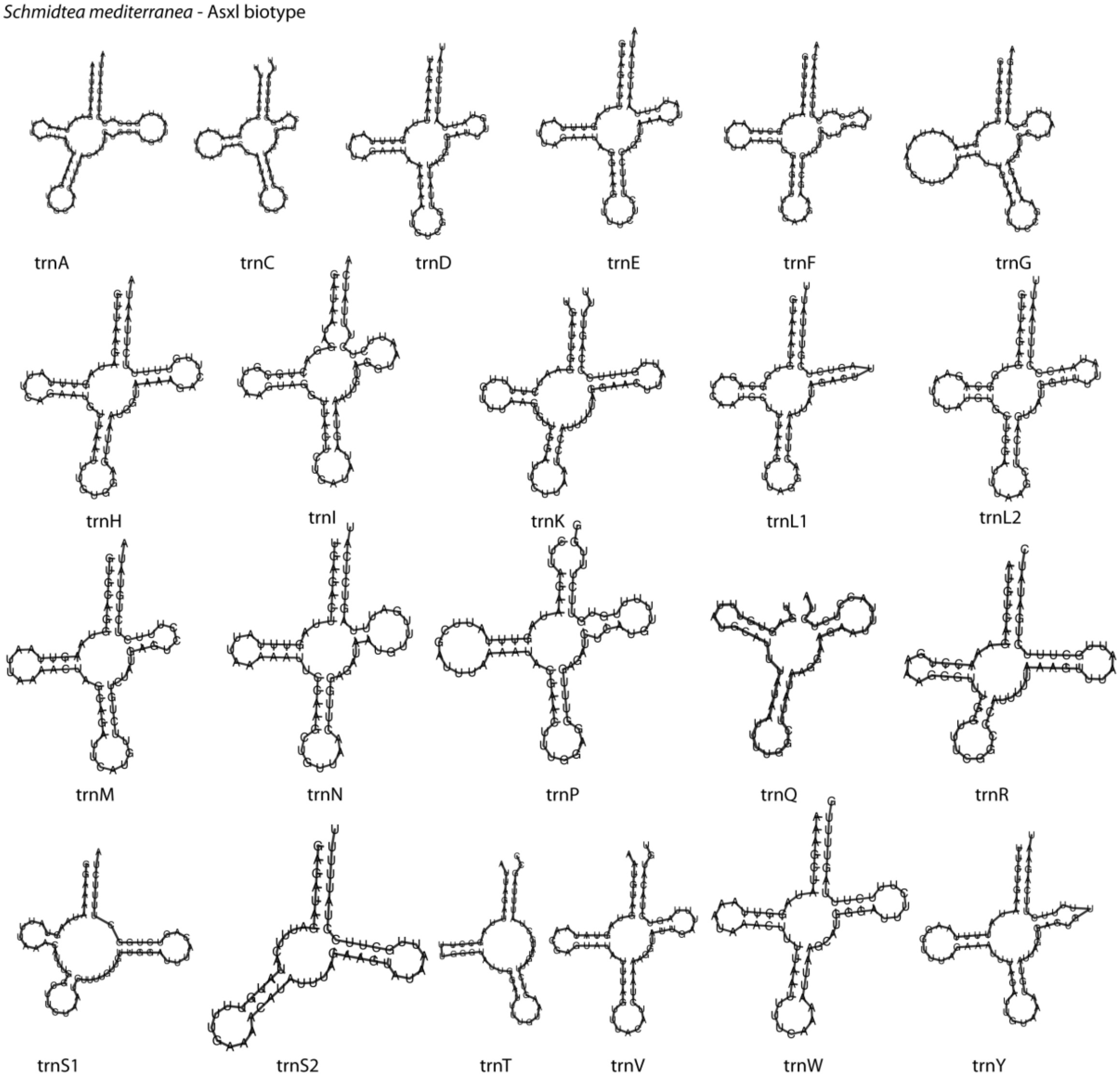

All tRNAs structures from MITOs
