# Supplementary figures and images for: Comparative and Transcriptome Analyses Uncover Key Aspects of Coding- and Long Noncoding RNAs in Flatworm Mitochondrial Genomes
Source: G3 (Bethesda). 2016 Feb 23;6(5):1191–200. doi: 10.1534/g3.116.028175 (PMC4856072; doi:10.1534/g3.116.028175)

**Figure S3 - Trna figures *Girardia* sp.**

tRNA secondary structures as predicted by MITOS.

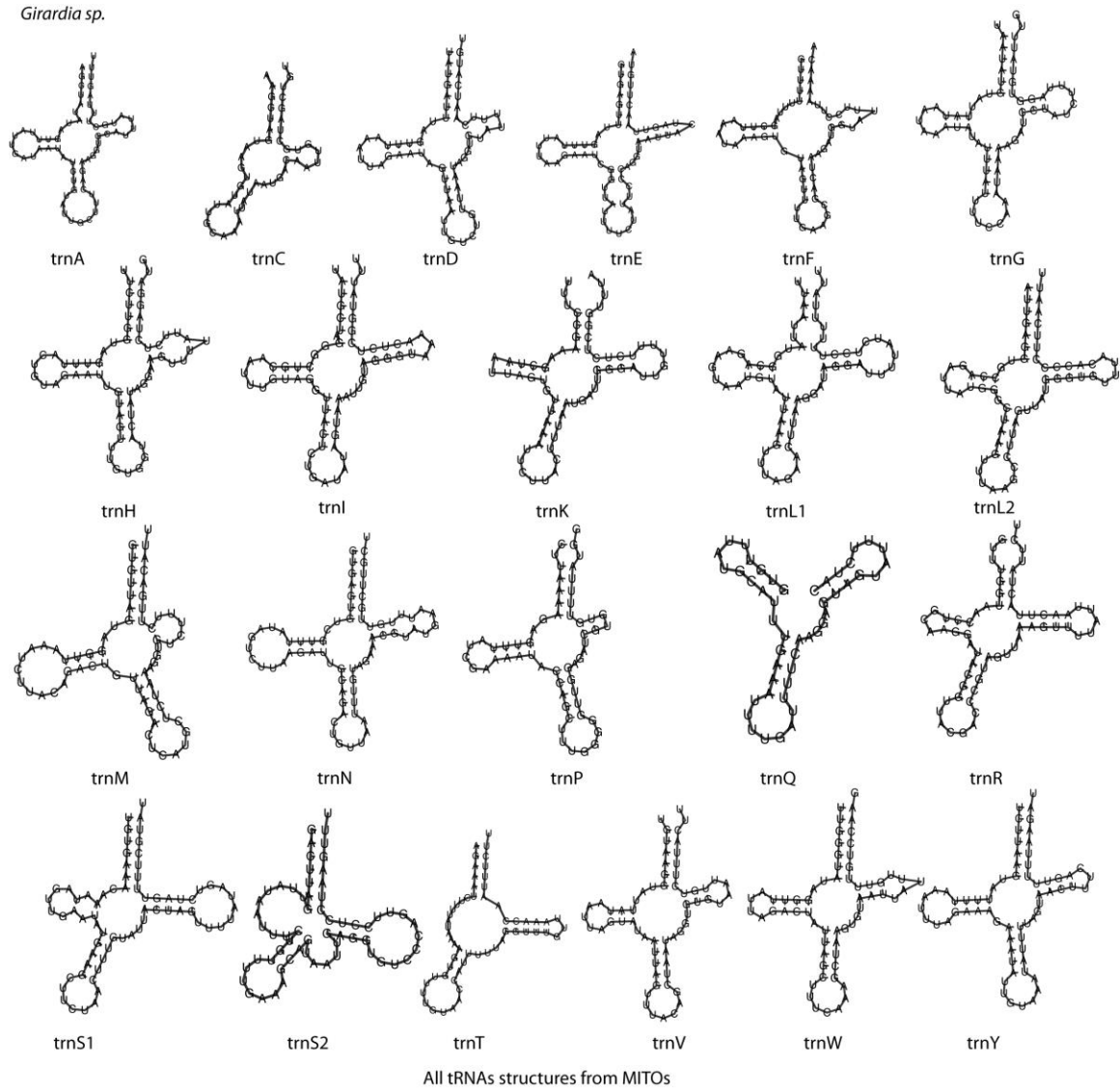

Supplement: Supplemental Material [file supp_g3.116.028175_FigureS3.pdf]
