# Supplementary material for: Comparative and Transcriptome Analyses Uncover Key Aspects of Coding- and Long Noncoding RNAs in Flatworm Mitochondrial Genomes
Source: G3 (Bethesda). 2016 Feb 23;6(5):1191–200. doi: 10.1534/g3.116.028175 (PMC4856072; doi:10.1534/g3.116.028175)
Supplement: Supplemental Material [file supp_g3.116.028175_FigureS5.pdf]

Figure S5 – Schematic of PCR and sequencing of key regions of the *S. mediterranea* mitochondrial genome.

A. SmedAsxl - Amplification Products

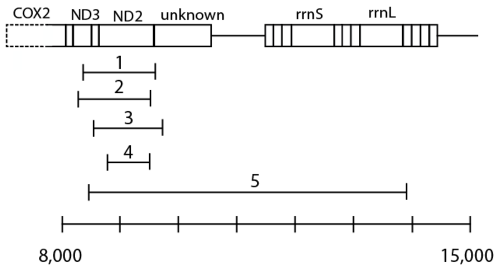

B. SmedSxl - Amplification Products

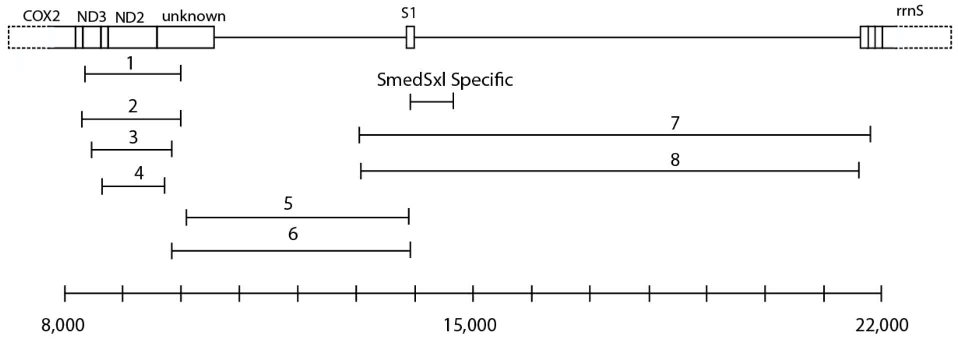

Non coding regions with surrounding genes for each *S. mediterranea* biotype. Numbered bars represent amplification primer locations and expected products. These numbers correspond to the numbers in Figure S6 and table S5. tRNA names are not show except for “S1”. See Figures 1 and 2 for full gene complement and tRNA names.
