# Supplementary material for: Comparative and Transcriptome Analyses Uncover Key Aspects of Coding- and Long Noncoding RNAs in Flatworm Mitochondrial Genomes
Source: G3 (Bethesda). 2016 Feb 23;6(5):1191–200. doi: 10.1534/g3.116.028175 (PMC4856072; doi:10.1534/g3.116.028175)
Supplement: Supplemental Material [file supp_g3.116.028175_FigureS6.pdf]

Figure S6. – Gel of PCR of key regions of the *S. mediterranea* mitochondrial genome. Fragments were sequenced and confirmed to correspond to predicted regions in sexual and asexual mitochondrial genome biotype assemblies.

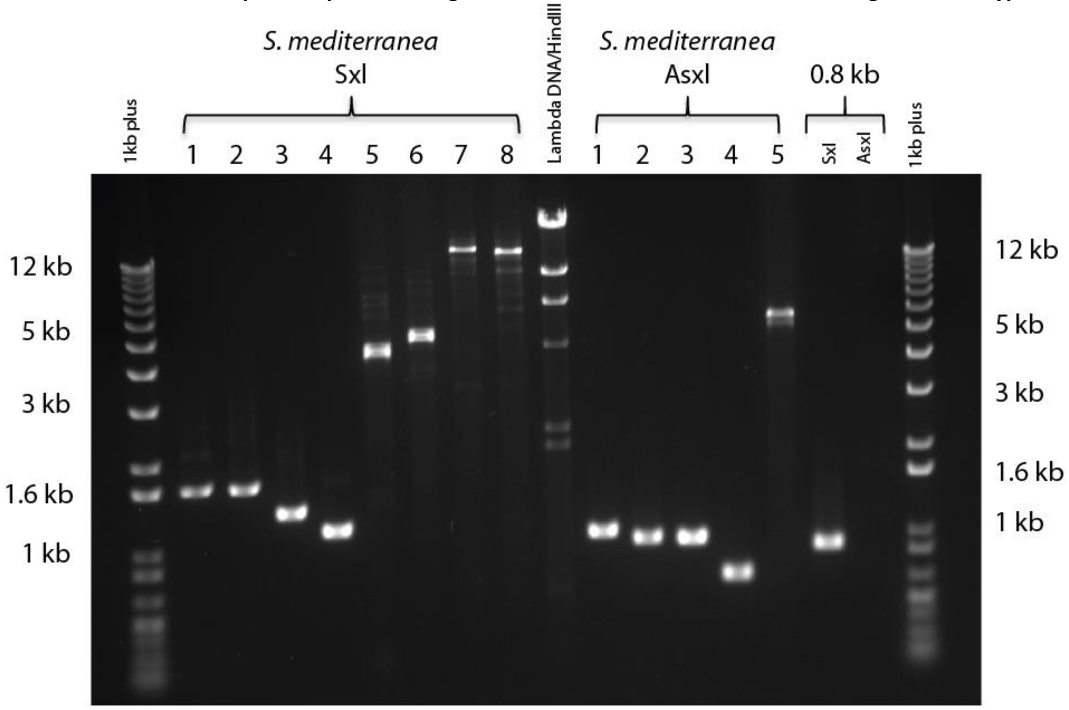

Gel showing product size for amplification products described in Table S5 and in Figure S5.
