# Supplementary material for: Comparative and Transcriptome Analyses Uncover Key Aspects of Coding- and Long Noncoding RNAs in Flatworm Mitochondrial Genomes
Source: G3 (Bethesda). 2016 Feb 23;6(5):1191–200. doi: 10.1534/g3.116.028175 (PMC4856072; doi:10.1534/g3.116.028175)
Supplement: Supplemental Material [file supp_g3.116.028175_TableS1.pdf]

Table S1 – SmedSxl Feature Table

Name	Start	Stop	Length	Distance	Putative Start Codon
COX1	427	2247	1821	427	ttg
trnE(ttc)	2336	2397	62	89	
ND6	2408	2854	447	11	atg
ND5	2848	4446	1599	-6	ttg
trnS2(tga)	4450	4508	59	4	
trnD(gtc)	4509	4570	62	1	
trnR(tcg)	4569	4632	64	-1	
COX3	4653	5456	804	21	ttg
trnI(gat)	5452	5515	64	-4	
trnQ(ttg)	5524	5572	49	9	
trnK(ctt)	5574	5642	69	2	
ATP6	5646	6281	636	4	atg
trnV(tac)	6283	6345	63	2	
ND1	6342	7232	891	-3	atg
trnW(tca)	7236	7300	65	4	
COX2	7301	8179	879	1	ttg
trnP(tgg)	8283	8353	71	104	
ND3	8354	8707	354	1	ttg
trnA(tgc)	8712	8780	69	5	
ND2	8781	9728	948	1	ttg
?	9728	10228	501	0	atg
trnS1(tct)	13897	13963	67	3669	
trnM(cat)	21440	21502	63	7477	
trnH(gtg)	21505	21571	67	3	
trnF(gaa)	21575	21640	66	4	
rrnS	21642	22348	707	2	
trnL1(tag)	22350	22412	63	2	
trnY(gta)	22418	22483	66	6	
trnG(tcc)	22489	22557	69	6	
rrnL	22558	23472	915	1	
trnL2(taa)	23473	23535	63	1	
trnT(tgt)	23536	23589	54	1	
trnC(gca)	23604	23663	60	15	
trnN(gtt)	23673	23736	64	10	
CYTB	24452	25546	1095	716	ttg
ND4L	25513	25806	294	-33	atg
ND4	25757	27133	1377	-49	atg
