# Supplementary material for: Comparative and Transcriptome Analyses Uncover Key Aspects of Coding- and Long Noncoding RNAs in Flatworm Mitochondrial Genomes
Source: G3 (Bethesda). 2016 Feb 23;6(5):1191–200. doi: 10.1534/g3.116.028175 (PMC4856072; doi:10.1534/g3.116.028175)
Supplement: Supplemental Material [file supp_g3.116.028175_TableS2.pdf]

**Table S2 – SmedAsxl Feature Table**

| Name | Start | Stop | Length | Distance | Putative Start Codon |
|---|---|---|---|---|---|
| COX1 | 428 | 2248 | 1821 | 427 | ttg |
| trnE(ttc) | 2337 | 2398 | 62 | 89 | |
| ND6 | 2409 | 2855 | 447 | 11 | atg |
| ND5 | 2849 | 4443 | 1595 | -6 | ttg |
| trnS2(tga) | 4450 | 4507 | 58 | 7 | |
| trnD(gtc) | 4508 | 4569 | 62 | 1 | |
| trnR(tcg) | 4568 | 4631 | 64 | -1 | |
| COX3 | 4652 | 5455 | 804 | 21 | ttg |
| trnI(gat) | 5451 | 5514 | 64 | -4 | |
| trnQ(ttg) | 5521 | 5573 | 53 | 7 | |
| trnK(ctt) | 5574 | 5642 | 69 | 1 | |
| ATP6 | 5643 | 6281 | 639 | 1 | atg |
| trnV(tac) | 6283 | 6345 | 63 | 2 | |
| ND1 | 6342 | 7232 | 891 | -3 | atg |
| trnW(tca) | 7236 | 7301 | 66 | 4 | |
| COX2 | 7302 | 8180 | 879 | 1 | ttg |
| trnP(tgg) | 8279 | 8349 | 71 | 99 | |
| ND3 | 8350 | 8703 | 354 | 1 | ttg |
| trnA(tgc) | 8708 | 8776 | 69 | 5 | |
| ND2 | 8777 | 9724 | 948 | 1 | ttg |
| ? | 9725 | 10225 | 501 | 1 | ttg |
| trnM(cat) | 11474 | 11536 | 63 | 1249 | |
| trnH(gtg) | 11541 | 11606 | 66 | 5 | |
| trnF(gaa) | 11610 | 11675 | 66 | 4 | |
| rrnS | 11676 | 12384 | 709 | 1 | |
| trnL1(tag) | 12385 | 12446 | 62 | 1 | |
| trnY(gta) | 12452 | 12517 | 66 | 6 | |
| trnG(tcc) | 12523 | 12591 | 69 | 6 | |
| rrnL | 12592 | 13506 | 915 | 1 | |
| trnL2(taa) | 13507 | 13570 | 64 | 1 | |
| trnT(tgt) | 13571 | 13624 | 54 | 1 | |
| trnC(gca) | 13639 | 13698 | 60 | 15 | |
| trnN(gtt) | 13707 | 13770 | 64 | 9 | |
| trnS1(tct) | 14409 | 14475 | 67 | 639 | |
| CYTB | 14497 | 15591 | 1095 | 22 | ttg |
| ND4L | 15558 | 15851 | 294 | -33 | atg |
| ND4 | 15802 | 17178 | 1377 | -49 | atg |
