# Supplementary material for: Comparative and Transcriptome Analyses Uncover Key Aspects of Coding- and Long Noncoding RNAs in Flatworm Mitochondrial Genomes
Source: G3 (Bethesda). 2016 Feb 23;6(5):1191–200. doi: 10.1534/g3.116.028175 (PMC4856072; doi:10.1534/g3.116.028175)
Supplement: Supplemental Material [file supp_g3.116.028175_TableS3.pdf]

Table S3 – *Girardia sp.* Feature Table

Name	Start	Stop	Length	Distance	Putative Start Codon
trnC(gca)	97	135	39	263	
trnN(gtt)	693	762	70	558	
CYTB	762	1892	1131	0	ttg
ND4L	1853	2146	294	-39	ttg
ND4	2115	3464	1350	-31	atg
COX1	3490	5304	1815	26	atg
trnE(ttc)	5311	5374	64	7	
ND6	5414	5845	432	40	ttg
ND5	5845	7422	1578	0	atg
trnS2(tga)	7527	7588	62	105	
trnD(gtc)	7589	7649	61	1	
trnR(acg)	7648	7710	63	-1	
COX3	7774	8565	792	64	ttg
trnI(gat)	8572	8638	67	7	
trnQ(ttg)	8646	8692	47	8	
trnK(ctt)	8695	8758	64	3	
ATP6	8775	9404	630	17	atg
trnV(tac)	9400	9461	62	-4	
ND1	9461	10348	888	0	ttg
trnW(tca)	10362	10423	62	14	
COX2	10424	11593	1170	1	ttg
trnP(tgg)	11589	11646	58	-4	
ND3	11686	11991	306	40	ttg
trnA(tgc)	11993	12054	62	2	
ND2	12058	12984	927	4	ttg
?	13024	13389	366	40	atg
trnM(cat)	13466	13529	64	77	
trnH(gtg)	13670	13733	64	141	
trnF(gaa)	13739	13799	61	6	
rrnS	13794	14514	721	-5	
trnL1(tag)	14507	14573	67	-7	
trnY(gta)	14573	14637	65	0	
trnG(tcc)	14640	14703	64	3	
trnS1(tct)	14702	14770	69	-1	
rrnL	14771	15663	893	1	
trnL2(taa)	15664	15727	64	1	
trnT(tgt)	15728	15785	58	1	
